# *PPARγ* Variant rs10865710 and Mortality in Pediatric Septic Shock Stratified by Corticosteroid Exposure

**DOI:** 10.1097/CCE.0000000000001410

**Published:** 2026-05-25

**Authors:** Valentina Bonnefil, Stephen Standage, Andrew J. Lautz, Natalja L. Stanski, Kelli Harmon, Patrick Lahni, Julie C. Fitzgerald, Adam J. Schwarz, Neal J. Thomas, Bereketeab Haileselassie, Basilia Zingarelli, Jennifer M. Kaplan, Mihir R. Atreya

**Affiliations:** 1 Division of Critical Care Medicine, Cincinnati Children’s Hospital Medical Center, Cincinnati, OH.; 2 Department of Pediatrics, University of Cincinnati College of Medicine, Cincinnati, OH.; 3 Division of Critical Care, Department of Anesthesiology and Critical Care, The University of Pennsylvania Perelman School of Medicine and Children’s Hospital of Philadelphia, Philadelphia, PA.; 4 Children’s Hospital of Orange County, Orange, CA.; 5 Penn State Hershey Children’s Hospital, Hershey, PA.; 6 Lucile Packard Children’s Hospital Stanford, Stanford University School of Medicine, Palo Alto, CA.

**Keywords:** corticosteroids, genetic variants, metabolism, pediatric septic shock, peroxisome proliferator-activated receptor

## Abstract

**OBJECTIVES:**

To determine whether genetic variation in peroxisome proliferator-activated receptor gamma (PPARγ) is associated with mortality across corticosteroid exposure strata in pediatric septic shock.

**DESIGN:**

Multicenter prospective observational study.

**SETTING:**

PICUs at multiple U.S. hospitals.

**PATIENTS:**

Children 1 week to 10 years old meeting consensus criteria for septic shock.

**INTERVENTIONS:**

None.

**MEASUREMENTS AND MAIN RESULTS:**

Genomic DNA was genotyped for two *PPARγ* single nucleotide variants (SNVs; rs10865710 and rs1801282) using TaqMan assays. Associations with 28-day mortality were evaluated using multivariable logistic regression and Cox proportional hazards models, with analyses stratified by systemic corticosteroid exposure within 72 hours. In a subset with whole-blood RNA sequencing, expression quantitative trait locus (eQTL) analyses assessed genotype effects on candidate transcripts. Among 381 patients, both SNVs were in Hardy-Weinberg equilibrium. Carriage of the rs10865710 mutant allele was associated with higher 28-day mortality (10.2% vs. 3.5%; *p* = 0.009), whereas rs1801282 showed no association. In stratified analyses, rs10865710 carriage was associated with increased mortality among corticosteroid-treated patients (adjusted odds ratio, 5.85; 95% CI, 1.62–30.44; *p* = 0.015) but not corticosteroid-naive patients. Similarly, in stratified Cox models, rs10865710 carriage was associated with increased hazard of death among corticosteroid-treated patients (hazard ratio, 5.33; 95% CI, 1.43–19.83; *p* = 0.013). Genotype was not associated with established mortality risk strata or transcriptomic endotype. In eQTL analyses (*n* = 81), rs10865710 carriage was not associated with PPARγ expression; however, a trend toward lower glucocorticoid receptor (*NR3C1*) expression was noted (*p* = 0.07).

**CONCLUSIONS:**

The intronic PPARγ variant rs10865710 is associated with increased mortality in pediatric septic shock, with the association most apparent among corticosteroid-treated patients. Although no cis-eQTL effect on *PPARγ* expression was detected, exploratory data suggest potential differences in glucocorticoid receptor signaling. Prospective validation and mechanistic studies are warranted.

**KEY POINTS**

**Question**: Is the *PPARγ* rs10865710 variant associated with mortality in pediatric septic shock, and does this association differ by corticosteroid exposure?

**Findings**: In a multicenter cohort of 381 children with septic shock, carriage of at least one rs10865710 mutant allele was associated with higher 28-day mortality. In stratified analyses, this association was observed among corticosteroid-treated patients but not among corticosteroid-naive patients.

**Meaning**: Inherited variation in *PPARγ* may contribute to outcome heterogeneity in pediatric septic shock, particularly in the setting of corticosteroid exposure. Because corticosteroid treatment was observational and not randomized, these findings should be interpreted as hypothesis-generating and warrant prospective validation.

Sepsis is a dysregulated host response to infection that can culminate in multiple organ dysfunction and death (1). Pediatric sepsis remains the leading cause of mortality among children under 5 years old worldwide (2, 3). Survivors of the acute phase frequently experience substantial morbidity, including new medical device dependence and long-term functional impairment (4). Despite this burden, current management remains limited to antimicrobial therapy and intensive organ support. More than 120 clinical trials have failed to identify an intervention that consistently improves outcomes in unselected populations (5), and the use of adjunctive corticosteroids in pediatric sepsis remains a subject of ongoing debate (6).

Peroxisome proliferator-activated receptors (PPARs) are ligand-activated nuclear receptors that, like glucocorticoids, regulate gene transcription and play central roles in metabolism and inflammation (7). While the metabolic functions of PPARs are well established, emerging evidence supports their involvement in immune cell differentiation and effector function, including in T cells and natural killer cells (8). Notably, PPARγ and glucocorticoids exert complementary yet distinct transcriptional control over inflammatory and metabolic programs (9), with glucocorticoids capable of inducing *PPARγ* expression and PPARγ modulating sensitivity to glucocorticoid signaling (10).

Prior work from our group demonstrated that children with septic shock exhibit reduced *PPARγ* expression and increased PPARγ activity in peripheral blood immune cells (11). However, whether genetic variation in PPARγ is associated with clinical outcomes in pediatric septic shock, particularly in the context of corticosteroid exposure, has not been examined. Accordingly, we genotyped patients in a large prospective pediatric septic shock cohort to test whether *PPARγ* single nucleotide variants (SNVs) were associated with clinical mortality across corticosteroid exposure strata. We further performed expression quantitative trait locus (eQTL) analyses to evaluate whether rs10865710 was associated with differences in whole-blood expression of *PPARγ* and *NR3C1* (encoding the glucocorticoid receptor). These analyses were designed to explore whether inherited variation in PPARγ contributes to outcome heterogeneity in pediatric septic shock.

## METHODS

### Human Participants and Data Collection

We used biospecimens and clinical data from an ongoing prospective cohort of critically ill children (12–14), 1 week to 10 years old, meeting international consensus criteria for septic shock (15). Patients were enrolled from multiple PICUs across the United States (Institutional Review Board [IRB] No. 2022-0710). The study was approved by the IRBs of participating institutions with Cincinnati Children’s Hospital Medical Center serving as the central IRB (continuing approval, most recently renewed January 20, 2026). The study was observational with no intervention. Informed consent was obtained from parents or legal guardians. There were no exclusion criteria other than inability to obtain consent.

- Whole blood was collected within 24 hours of PICU admission. De-identified clinical data were collected daily from days 1–7 and included illness severity, therapies, and organ failure assessments. Illness severity was quantified using the Pediatric Risk of Mortality (PRISM) III score (16) at PICU admission. Mortality was assessed through 28 days after enrollment. Data were entered into a centralized, encrypted Research Electronic Data Capture database (Vanderbilt University, Nashville, TN), and curated datasets were maintained on LabKey (LabKey Software, Seattle, WA). The primary outcome was 28-day mortality. Secondary outcomes included: 1) complicated course, defined as persistence of greater than or equal to 2 organ failures at day 7 or death within 28 days (12), 2) PICU length of stay, and 3) PICU-free days.

Corticosteroid administration was at the discretion of treating physicians. Exposure variables captured receipt of any systemic corticosteroid within 72 hours of enrollment, including hydrocortisone administration. For primary analyses, corticosteroid exposure was modeled as a binary variable (any systemic corticosteroid within 72 hr). Stratified analyses were performed according to corticosteroid exposure status. Detailed information regarding cumulative dose, duration of therapy, precise timing relative to shock onset, and cortisol testing was not available.

### Genotyping

Genomic DNA was extracted from whole blood using standard protocols. DNA concentration was measured by NanoDrop spectrophotometry (Thermo Fisher Scientific, Waltham, MA), and samples were diluted to 4 ng/μL in DNase-free water. Genotyping was performed using TaqMan 5′ nuclease assays (Thermo Fisher Scientific, Waltham, MA) on the QuantStudio 6 Flex Real-Time Polymerase Chain Reaction platform (Thermo Fisher Scientific, Waltham, MA). Commercially available primer-probe sets were used for *PPARγ* intronic SNV rs10865710 and coding SNV rs1801282, both of which have reported minor allele frequencies between 5% and 25%. These variants were prespecified a priori based on prior literature implicating them in sepsis susceptibility and immunometabolic regulation and were selected for their biologic plausibility rather than derived from a broader discovery screen.

### Mortality Risk Stratification and Endotypes

Molecular risk and endotype classifications were available for a significant proportion of participants included in the study. Mortality risk strata were defined using the pediatric sepsis biomarker risk mode (PERSEVERE) biomarker-based model (12, 17), which integrates serum concentrations of multiple immune proteins to generate validated mortality risk estimates in pediatric septic shock (17). Additionally, transcriptomic endotypes were assigned based on NanoString nCounter gene expression profiling (NanoString Technologies, Seattle, WA), using a previously established 100-gene classifier that distinguishes endotype A and B patterns (13). These endotypes have been shown to capture biologically distinct host-response states and have been associated with differential treatment responses to corticosteroids (13).

### Protein Biomarkers

Serum immune (17) and endothelial protein biomarkers (14, 18) were previously quantified in a substantial portion of patients using Luminex Multiplex assays (R&D Systems, Minneapolis, MN), as described in prior publications.

### Gene Expression Data

Whole-blood bulk messenger RNA sequencing data were available in a subset of the dataset (18). RNA sequencing libraries were prepared from PAXgene-preserved whole blood, normalized to correct for batch effects, and expressed as log-transformed counts per million. Targeted genes of interest were extracted for secondary analyses.

### Statistical Analyses

All analyses were performed using R (Version 4.3.2; R Foundation, Vienna, Austria). Hardy-Weinberg equilibrium for each SNV was evaluated using the chi-square test. Baseline variables were summarized as frequencies for categorical data and medians with interquartile ranges for continuous data. Between-group comparisons used chi-square or Fisher exact tests for categorical variables and Wilcoxon rank-sum tests for continuous variables.

Genotype was analyzed under a dominant genetic model (wildtype vs. ≥ 1 minor allele carrier) for all primary and secondary analyses. Associations between rs10865710 genotype and 28-day mortality were evaluated using multivariable logistic regression, stratified by systemic corticosteroid exposure within 72 hours of enrollment. Prespecified covariates included age, sex, self-reported race, PRISM III score, and presence of any comorbidity. Given the limited number of deaths, the number of covariates was restricted to reduce overfitting, and genotype was retained in all models regardless of statistical significance.

Time-to-event analyses were conducted using Cox proportional hazards models to evaluate associations with 28-day survival, similarly, stratified by corticosteroid exposure and adjusted for age, sex, self-reported race, PRISM III score, and comorbidity status. The proportional hazards assumption was assessed using Schoenfeld residuals.

### Expression Quantitative Trait Locus Analysis

eQTL analyses were conducted in patients with available day 1 whole-blood RNA sequencing data. Associations between rs10865710 genotype and log-transformed PPARγ and NR3C1 expression were evaluated using linear regression under a dominant genetic model (wildtype vs. ≥ 1 minor allele). All models were adjusted for age, sex, self-reported race, PRISM III score, and comorbidity status; models additionally adjusted for corticosteroid exposure are specified. Robust ses were used to account for heteroskedasticity consistent type 3. Given the limited sample size of patients with paired genotype and transcriptomic data, these analyses were exploratory and not powered to detect modest effect sizes. No correction for multiple comparisons was applied.

## RESULTS

A total of 410 pediatric patients with septic shock were genotyped. After exclusion of samples with inadequate quality, genotyping failure, or missing clinical data, 381 patients were included in the final analysis (**Fig. S1**, https://links.lww.com/CCX/B628). PPARγ SNVs tested were in Hardy-Weinberg equilibrium (rs10865710: χ^2^ = 0.592; rs1801282: χ^2^ = 0.742; **Supplementary Table 1**, https://links.lww.com/CCX/B628). Among these, 178 patients carried at least one copy of the rs10865710 mutant G allele, and 80 patients carried mutant alleles of rs1801282. Baseline illness severity and comorbid conditions did not differ significantly between carriers and noncarriers of either variant.

Patients carrying greater than or equal to 1 copy of the mutant rs10865710 allele had higher 28-day mortality compared with those without (10.2% [18/178] vs. 3.5% [7/203]; *p* = 0.009). Although numerically higher, the proportion of patients with a complicated course did not differ significantly by genotype (*p* = 0.071; **Table 1**). Among rs1801282 mutant carriers, a higher proportion self-identified as White/Caucasian compared with the wildtype group (86.3% vs. 71.1%; *p* < 0.001). However, this SNV was not associated with differences in 28-day mortality or secondary outcomes (**Supplementary Table 2**, https://links.lww.com/CCX/B628).

**TABLE 1.** Demographic Data and Clinical Outcomes According to Presence of At Least One Copy of the *PPARγ* rs10865710 Mutant Allele

Variable	Wildtype (*n* = 203)	Mutant (*n* = 178)	*p*
Age (yr)	3.5 (1.2–7.0)	2.8 (1.0–6.0)	0.094
Sex (female)	96 (47.3%)	79 (44.4%)	0.570
Self-reported race			0.747
White/Caucasian	151 (74.4%)	132 (74.2%)	
Black/African American	30 (14.8%)	23 (12.9%)	
Other	22 (10.9%)	23 (12.9%)	
Ethnicity (Hispanic/Latino)	24 (11.9%)	31 (17.4%)	0.213
Pediatric Risk of Mortality III	11 (6–16)	11 (7–17)	0.526
Comorbidity (yes)	47 (23.2%)	49 (27.5%)	0.326
Adrenal insufficiency	6 (3.1%)	4 (2.3%)	0.658
Immunosuppression	20 (9.9%)	22 (12.4%)	0.436
Bone marrow transplantation	6 (2.9%)	6 (3.4%)	0.817
Corticosteroids (yes)	96 (47.3%)	87 (48.9%)	0.757
Day 1 hydrocortisone	65 (33.0%)	53 (30.5%)	0.601
Days 1–3 hydrocortisone	75 (38.1%)	65 (37.4%)	0.887
28-d mortality	7 (3.5%)	18 (10.2%)	0.009
Complicated course	39 (19.3%)	48 (27.1%)	0.071

Data are presented as median (interquartile range) or *n* (%).

**Figure 1.**
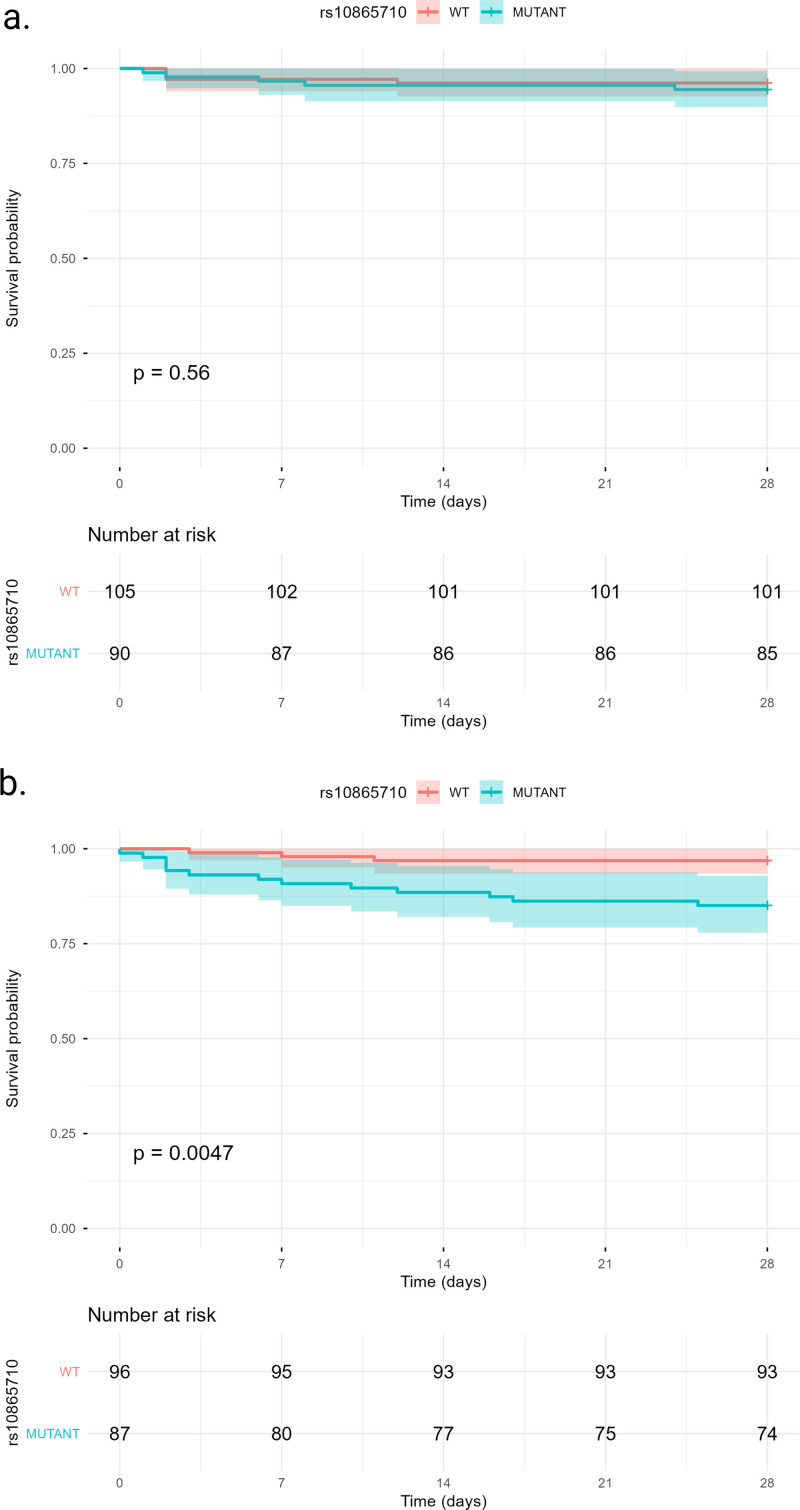
Survival analyses stratified by peroxisome proliferator-activated receptor gamma single nucleotide variants and corticosteroid exposure. Kaplan-Meier survival curves and number at risk according to presence of at least one mutant allele of *PPARγ* single nucleotide variant rs10865710 in (**A**) corticosteroid-naive and (**B**) corticosteroid-treated patients. WT = wildtype.

In multivariable logistic regression stratified by corticosteroid exposure, rs10865710 carriage was associated with increased mortality among corticosteroid-treated patients (adjusted odds ratio, 5.85; 95% CI, 1.62–30.44; *p* = 0.015), but not among corticosteroid-naive patients (**Table 2**). Kaplan-Meier survival curves similarly demonstrated reduced survival among corticosteroid-treated rs10865710 carriers (log-rank *p* = 0.0047), whereas no significant survival difference was observed in corticosteroid-naive patients (log-rank *p* = 0.56; **Fig. 1**). In adjusted Cox proportional hazards models stratified by corticosteroid exposure, rs10865710 carriage was associated with an increased hazard of death among corticosteroid-treated patients (hazard ratio; 5.33; 95% CI, 1.43–19.83; *p* = 0.013), but not among corticosteroid-naive patients (**Table 3**). No significant association was observed between rs1801282 genotype and mortality (**Supplementary Tables 3** and **4**, https://links.lww.com/CCX/B628).

**TABLE 2.** Multivariable Logistic Regression Testing the Association Between *PPARγ* Single Nucleotide Variant rs10865710 and 28-Day Mortality Stratified by Corticosteroid Exposure

Variable	OR (95% CI)	*p*
Corticosteroid naive		
Age (per unit)	0.83 (0.58–1.11)	0.252
Sex (female vs. male)	0.16 (0.01–1.03)	0.105
Race (Black vs. White)	0.98 (0.05–8.06)	0.986
Race (other vs. White)	0.65 (0.03–5.80)	0.737
Any comorbidity (yes vs. no)	4.37 (0.81–23.77)	0.078
rs10865710 mutant (mutant vs. wildtype)	**1.23 (0.26–5.86**)	**0.788**
Corticosteroid treated		
Age (per unit)	0.92 (0.74–1.10)	0.386
Sex (female vs. male)	1.31 (0.40–4.37)	0.651
Race (Black vs. White)	0.86 (0.12–4.06)	0.861
Race (other vs. White)	1.71 (0.23–8.32)	0.541
Any comorbidity (yes vs. no)	3.16 (1.01–10.44)	0.050
rs10865710 mutant (mutant vs. wildtype)	**5.85 (1.62–30.44**)	**0.015**

Bolded rows indicate the prespecified genotype comparison of interest, rs10865710 mutant carrier status versus wildtype status, within each corticosteroid exposure stratum. Other rows show covariates included for model adjustment and are provided to describe the fitted model; these covariate estimates should not be interpreted as independent effects.

OR = odds ratio.

**TABLE 3.** Cox Proportional Hazards Regression Testing Effect of *PPARγ* rs10865710 Mutant on 28-Day Survival Time Stratified by Corticosteroid Exposure

Variable	Hazard Ratio (95% CI)	*p*
Corticosteroid naive		
Age (per unit)	0.87 (0.65–1.15)	0.326
Sex (female vs. male)	0.19 (0.02–1.53)	0.118
Race (Black vs. White)	0.93 (0.10–8.94)	0.948
Race (other vs. White)	0.83 (0.09–7.95)	0.872
Any comorbidity (yes vs. no)	2.84 (0.62–12.95)	0.177
rs10865710 mutant (mutant vs. wildtype)	**0.98 (0.24–4.06**)	**0.980**
Corticosteroid treated		
Age (per unit)	0.90 (0.75–1.08)	0.258
Sex (female vs. male)	1.60 (0.52–4.96)	0.411
Race (Black vs. White)	0.91 (0.20–4.12)	0.906
Race (other vs. White)	1.49 (0.32–7.04)	0.612
Any comorbidity (yes vs. no)	2.76 (0.98–7.81)	0.055
rs10865710 mutant (mutant vs. wildtype)	**5.33 (1.43–19.83**)	**0.013**

Bolded rows indicate the prespecified genotype comparison of interest, rs10865710 mutant carrier status versus wildtype status, within each corticosteroid exposure stratum. Other rows show covariates included for model adjustment and are provided to describe the fitted model; these covariate estimates should not be interpreted as independent effects.

There was no significant overlap between carriers of greater than or equal to 1 rs10865710 mutant allele and previously defined pediatric septic shock gene expression endotypes (A vs. B) that have demonstrated differential corticosteroid responsiveness (χ^2^ = 0.096; Cramer’s V = 0.094). Similarly, genotype was not associated with protein biomarker-based mortality risk strata (χ^2^ = 0.616; Cramer’s V = 0.051; **Fig. S2**, https://links.lww.com/CCX/B628). Next, we examined whether circulating immune and endothelial biomarkers differed by rs10865710 genotype and corticosteroid exposure. None of the nine biomarkers tested demonstrated significant genotype-by-corticosteroid interaction effects (all interaction *p* > 0.05; **Fig. S3**, https://links.lww.com/CCX/B628).

**Figure 2.**
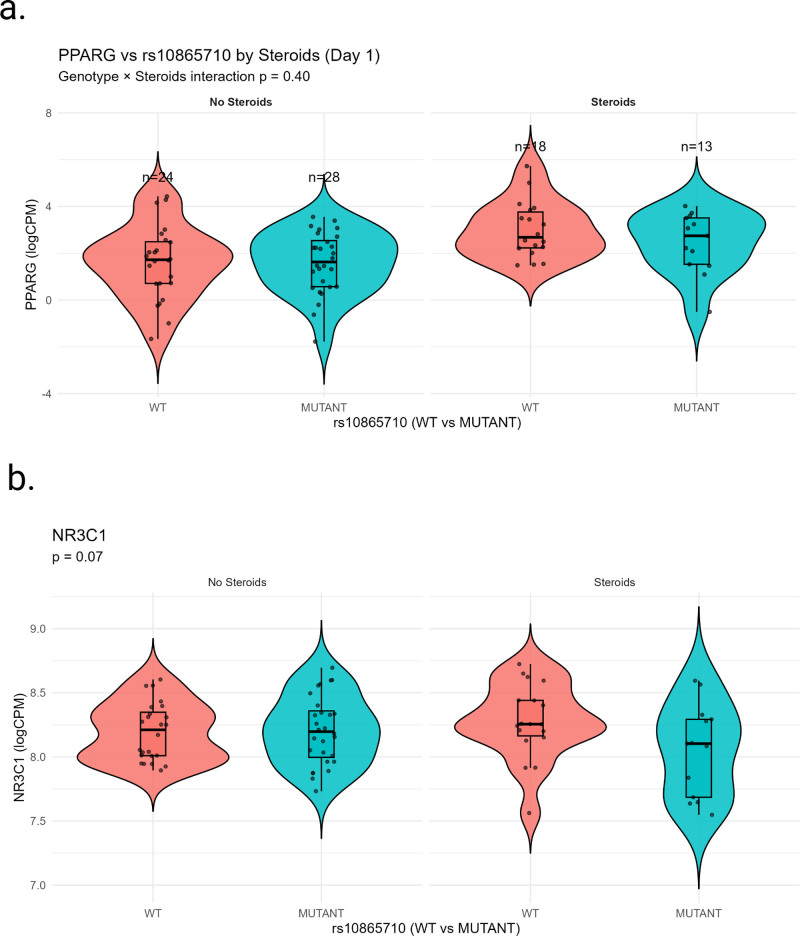
Twenty-eight-day survival stratified by *PPARγ* rs10865710 genotype and corticosteroid exposure. Whole blood (**A**) *PPARγ* and (**B**) *NR3C1* gene expression by single nucleotide variant rs10865710 and receipt of corticosteroids. *p values represent genotype × corticosteroid interaction terms from covariate-adjusted linear regression models (age, sex, race, comorbidity, Pediatric Risk of Mortality III; heteroskedasticity consistent type 3—a robust measure of SE). logCPM = log-transformed counts per million, WT = wildtype.

Whole-blood transcriptomic data were available for 81 children with corresponding genotype and clinical data on day 1. Corticosteroid exposure did not differ by rs10865710 genotype (χ^2^ = 1.07; *p* = 0.59). Under the dominant genetic model, rs10865710 carriage was not associated with *PPARγ* transcript abundance in models adjusted for age, sex, race, comorbidity status, and PRISM III score (β = 0.04 ± 0.12; *p* = 0.74), or in models additionally adjusted for corticosteroid exposure (β = 0.03 ± 0.14; *p* = 0.83; **Table 4**). Similarly, rs10865710 carriage was not associated with *NR3C1* expression in covariate-adjusted models without corticosteroid adjustment (β = 0.10 ± 0.41; *p* = 0.81) or with additional adjustment for corticosteroid exposure (β = 0.12 ± 0.43; *p* = 0.78). However, a genotype × corticosteroid interaction term demonstrated a nonsignificant trend for *NR3C1* expression (interaction *p* = 0.07; **Fig. 2**).

**TABLE 4.** Association of rs10865710 Genotype With Whole-Blood *PPARγ* and *NR3C1* Expression (Expression Quantitative Trait Locus Analysis)

Gene	Adjusted for Corticosteroids	Regression Coefficient β ± Robust se	95% CI (Lower–Upper)	*t* Statistic	*p*
*PPARγ*	No	0.040 ± 0.124	–0.202 to 0.283	0.325	0.74
	Yes	0.029 ± 0.135	–0.236 to 0.293	0.213	0.83
*NR3C1*	No	0.098 ± 0.408	–0.702 to 0.899	0.241	0.81
	Yes	0.122 ± 0.425	–0.711 to 0.955	0.287	0.78

Linear regression models were used to evaluate the association between rs10865710 genotype and whole-blood *PPARγ* log-transformed counts per million under a dominant genetic model (wildtype vs. ≥ 1 mutant allele). Analyses were restricted to day 1 samples. All models were adjusted for age, sex, self-reported race, comorbidity status, and Pediatric Risk of Mortality III score; models additionally adjusted for corticosteroid exposure are indicated. Robust ses were used to account for heteroskedasticity consistent type 3.

## DISCUSSION

In this multicenter cohort of children with septic shock, carriage of greater than or equal to 1 mutant allele of the intronic PPARγ variant rs10865710 was associated with higher 28-day mortality. This association was observed among corticosteroid-treated patients but not among corticosteroid-naive patients. Because corticosteroid administration was not randomized, these findings should be interpreted as observational associations rather than evidence of a causal genotype-treatment interaction. In contrast, the missense coding variant rs1801282 was not associated with mortality or secondary outcomes.

The association between rs10865710 carriage and mortality persisted after adjustment for illness severity and comorbidity status and was not explained by established biomarker-mortality risk strata or transcriptomic endotype classification. In addition, circulating immune and endothelial biomarker profiles did not demonstrate significant genotype-by-corticosteroid interaction effects. Together, these findings suggest that the observed association is not attributable to measured baseline risk classification or broad inflammatory biomarker differences and instead may reflect a source of biological heterogeneity not captured by existing risk stratification tools.

In the subset of patients with available whole-blood transcriptomic data, rs10865710 carriage was not associated with PPARγ transcript abundance in covariate-adjusted models, arguing against a detectable cis-eQTL effect in this cohort. Similarly, no main-effect association was observed between rs10865710 carriage and *NR3C1* expression in adjusted models. However, a nonsignificant genotype × corticosteroid interaction was observed for NR3C1 expression (interaction *p* = 0.07). Although exploratory and underpowered, this finding raises the possibility that variation at this locus may relate to glucocorticoid receptor signaling. Whether this reflects altered receptor regulation, downstream transcriptional sensitivity, or statistical variability cannot be determined from these data.

Prior studies have linked PPARγ variants to sepsis susceptibility (19), including associations involving rs10865710 in adult trauma-related sepsis cohorts (20). Our results extend this literature by evaluating pediatric septic shock outcomes and by examining associations within corticosteroid exposure strata. These findings generate testable hypotheses regarding whether inherited variation in immunometabolic pathways may contribute to heterogeneity in outcomes in the context of corticosteroid treatment, but as such do not establish causality.

Several limitations warrant consideration. Corticosteroid administration was clinician-directed and therefore subject to confounding by indication. Although genotype groups did not differ in corticosteroid exposure and models adjusted for illness severity and comorbidity, residual confounding remains possible. Detailed information regarding cumulative steroid dose, timing relative to shock onset, and cortisol testing was not available. Population stratification may also influence genotype-outcome associations because ancestry-informative genetic markers were not included. Finally, transcriptomic analyses were limited to whole blood and a subset of patients, reducing power and limiting cell-type–specific inference. Collectively, these constraints underscore that the observed associations should be considered hypothesis-generating.

## CONCLUSIONS

In this prospective multicenter cohort of pediatric septic shock, carriage of the intronic *PPARγ* rs10865710 mutant allele was associated with increased mortality, with the association observed among corticosteroid-treated patients in stratified analyses. No cis-eQTL effect on PPARγ expression was detected, and transcriptomic findings were exploratory. Prospective studies incorporating mechanistic validation and protocolized corticosteroid administration are needed to determine whether genotype-informed risk stratification or therapeutic strategies have clinical relevance.

## ACKNOWLEDGMENTS

The Sepsis Genomics Collaborative members are as follows: Natalie Z. Cvijanovich (UCSF Benioff Children’s Hospital Oakland, Oakland, CA); Scott L. Weiss (Nemours Children’s Health, Wilmington, DE); Parag N. Jain (UT Southwestern Medical Center, Dallas, TX); Riad Lutfi (Riley Hospital for Children, Indianapolis, IN); and Jocelyn R. Grunwell (Children’s Healthcare of Atlanta at Egleston, Atlanta, GA).

## Supplementary Material
