# Supplementary material for: PPARγ Variant rs10865710 and Mortality in Pediatric Septic Shock Stratified by Corticosteroid Exposure
Source: Crit Care Explor. 2026 May 25;8(6):e1410. doi: 10.1097/CCE.0000000000001410 (PMC13201038; doi:10.1097/CCE.0000000000001410)
Supplement: Supplementary file 1 [file cc9-8-e1410-s001.pdf]

Online Supplement:

PPARγ Variant rs10865710 and Mortality in Pediatric Septic Shock Stratified by Corticosteroid Exposure

Authors: Valentina Bonnefil[1]; Stephen Standage[1,2]; Andrew J. Lautz[1,2]; Natalja L. Stanski[1,2]; Kelli Harmon[1]; Patrick Lahni[1]; Julie C. Fitzgerald[3]; Adam J. Schwarz[4]; Neal J. Thomas[5]; Bereketeab Haileselassie[6]; Basilia Zingarelli[1,2;] Jennifer M. Kaplan[1,2]; and Mihir R. Atreya[1,2], for the Sepsis Genomics Collaborative.

Affiliations:

1. Division of Critical Care Medicine, Cincinnati Children's Hospital Medical Center, Cincinnati, OH, 45229, USA.
2. Department of Pediatrics, University of Cincinnati College of Medicine, Cincinnati, OH, 45627, USA.
3. Division of Critical Care, Department of Anesthesiology and Critical Care, The University of Pennsylvania Perelman School of Medicine and Children's Hospital of Philadelphia, Philadelphia, PA 19104, USA
4. Children's Hospital of Orange County, Orange, CA 92868, USA
5. Penn State Hershey Children's Hospital, Hershey, PA 17033, USA
6. Lucile Packard Children's Hospital Stanford, Stanford University School of Medicine, Palo Alto, CA 94304, USA

Corresponding Author:
Mihir R Atreya, MD, MPH
Cincinnati Children's Hospital Medical Center
Division of Critical Care Medicine, MLC2005
3333 Burnet Avenue
Cincinnati, OH, 45229, USA
Tel: 513-636-1627
Email: Mihir.Atreya@cchmc.org

Short Title: *PPARγ* genetic variants in Pediatric Septic Shock.

Figure S1: Flow diagram of patients included in the study.

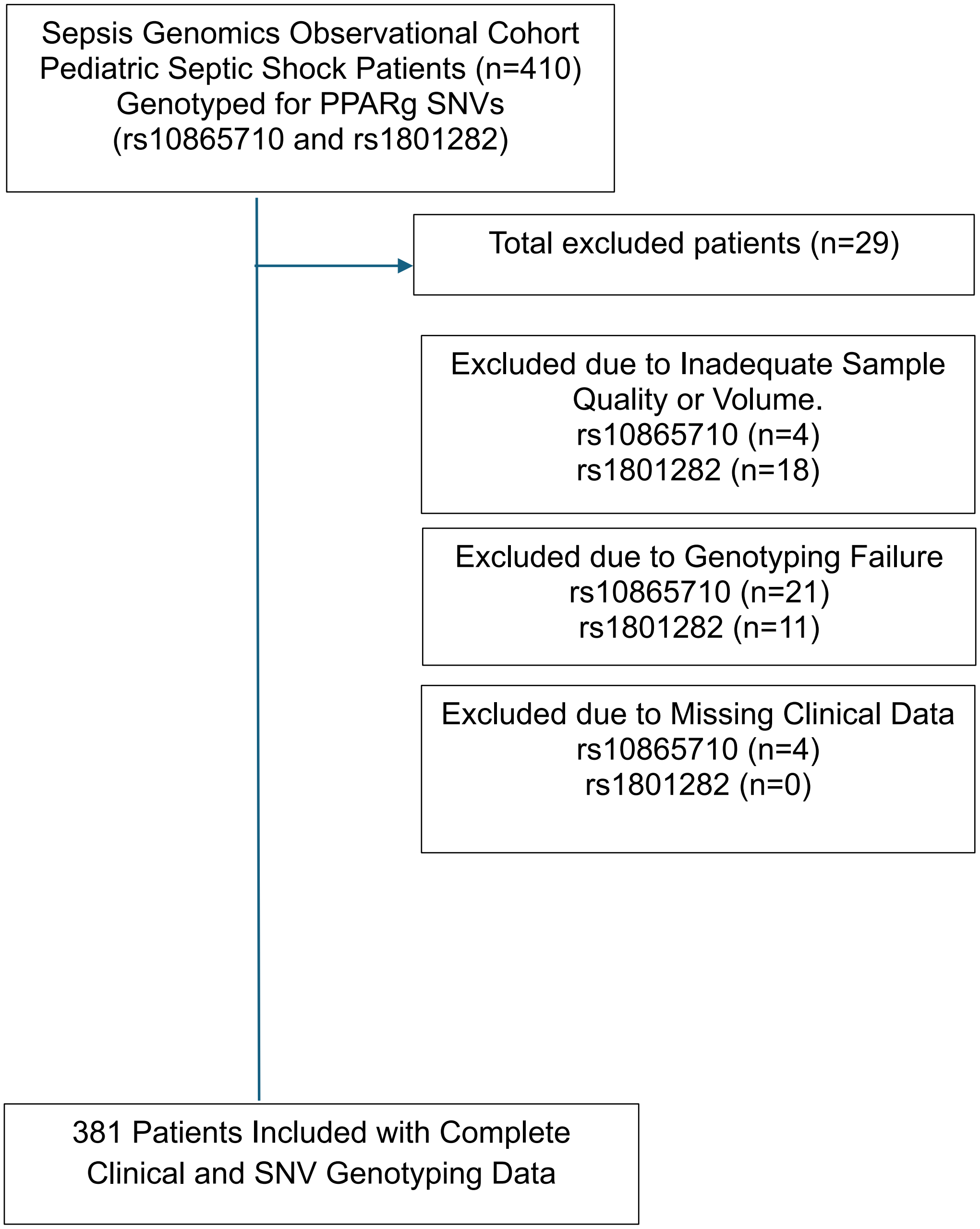

*Variant-specific counts reflect assay-level exclusions and may overlap within the same individuals

Figure S2: Alluvial Plots showing overlaps between *PPARγ* SNV rs10865710 and pediatric septic shock endotype and biomarker mortality risk strata.

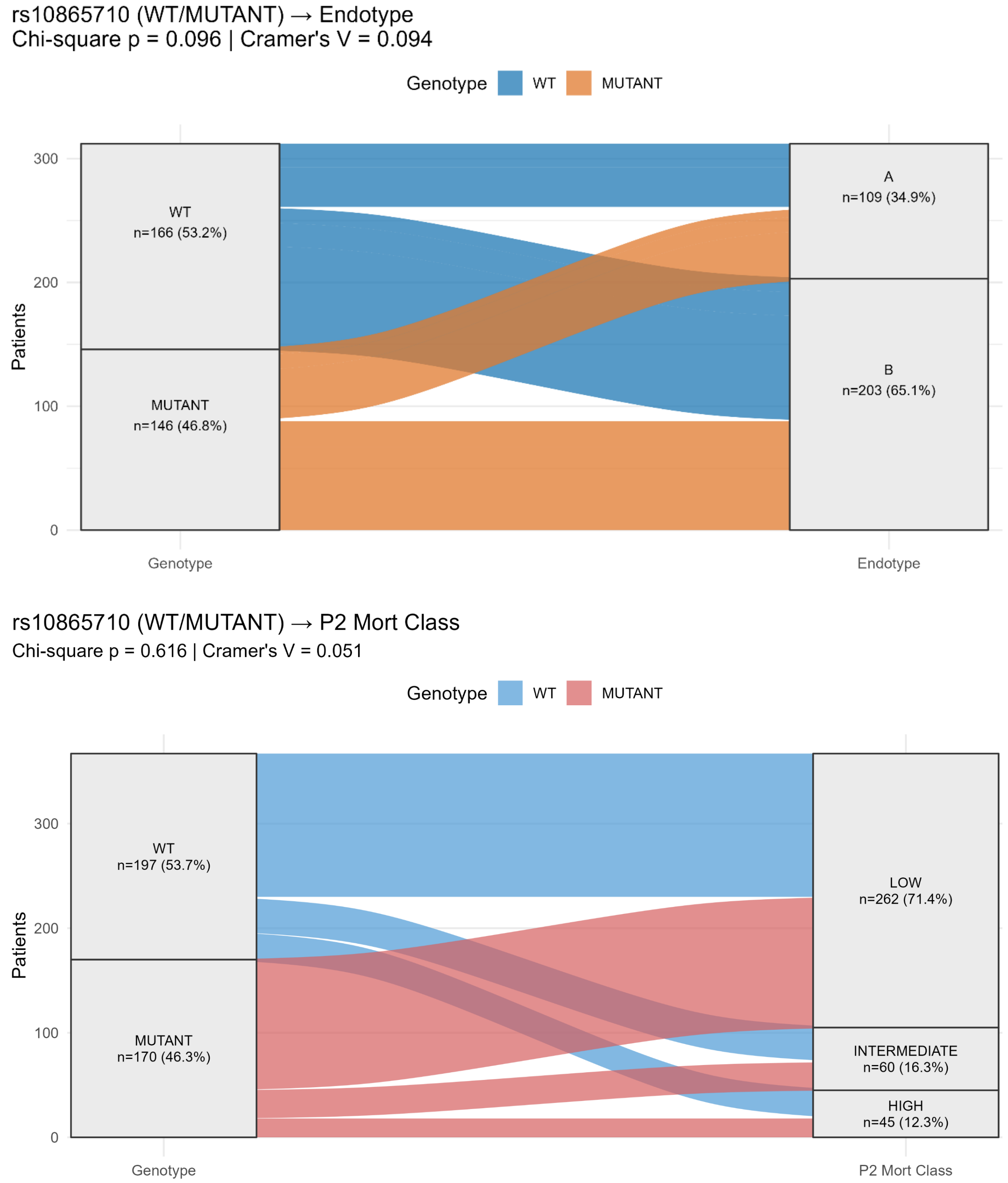

Figure S3: Immune and endothelial biomarkers according to *PPARγ* SNV rs10865710 and receipt of corticosteroids.

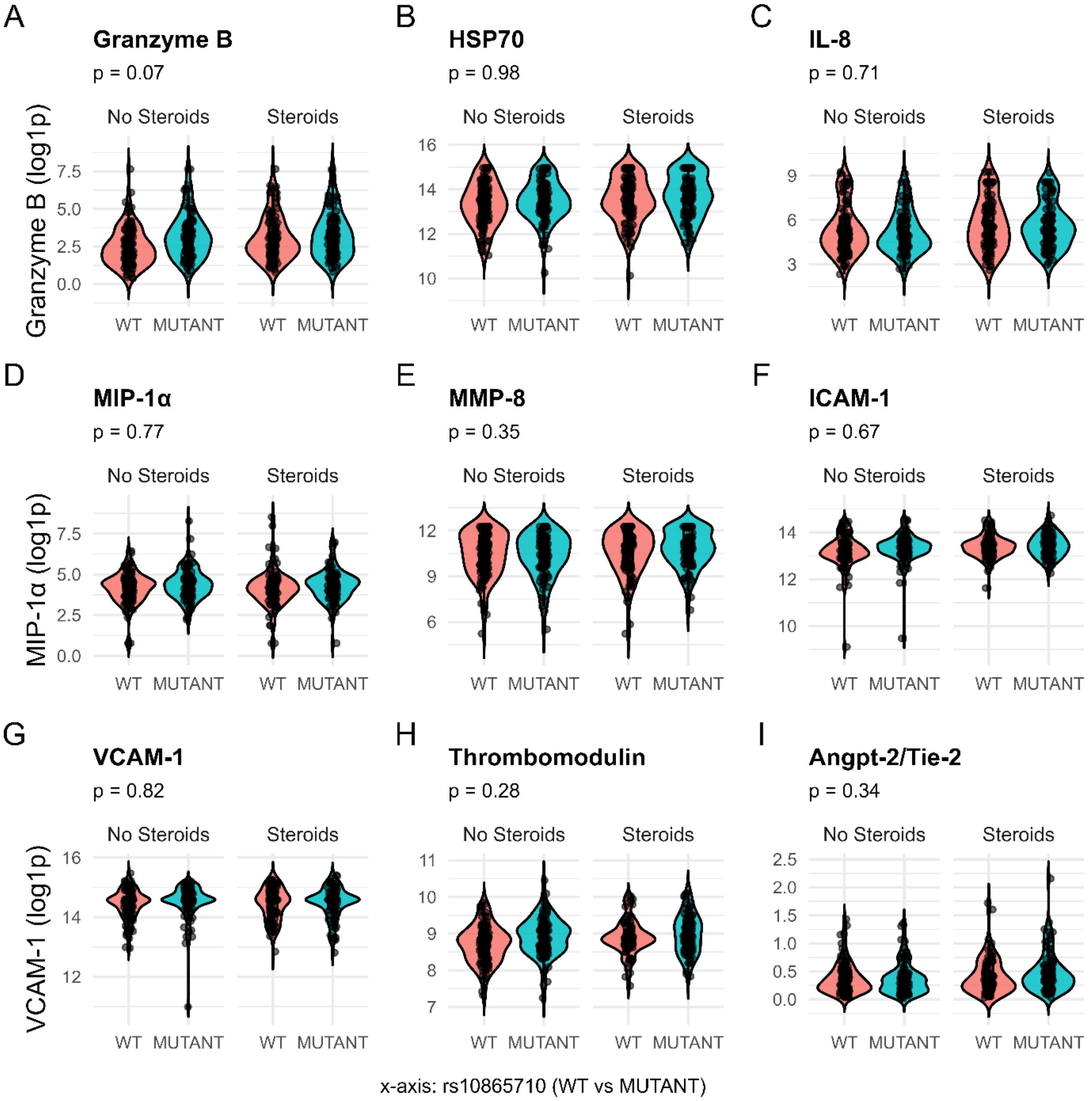

P values indicate interaction p values for genotype X receipt of corticosteroids effect on biomarker concentrations.

Supplementary Table 1: Hardy Weinberg Equilibrium (HWE) Testing between *PPARγ* SNVs among pediatric septic shock patients in the cohort, stratified by self-reported race.

	**Wildtype (C/C)**	**Heterozygous Mutant (C/G)**	**Homozygous Mutant (G/G)**	**MAF**	**HWE χ^2 p value**
**Rs10865710**	203	153	25	0.26	0.592
White	151	112	20	0.27	0.901
Black	30	21	2	0.24	0.470
Other	22	20	3	0.28	0.583
**Rs1801282**	301	76	4	0.11	0.742
White	214	65	4	0.13	0.708
Black	52	1	0	0.01	0.944
Other	35	10	0	0.11	0.401

MAF-Minor Allelic Frequency

Supplementary Table 2: Demographic data and clinical outcomes according to presence of at least one copy of the *PPARγ* rs1801282 mutant allele.

	Rs1801282 WT (n=301)	Rs1801282 Mutant (n=80)	P-value
Age (Years)	3.4 (1.1, 6.6)	2.4 (1.1, 5.4)	0.166
Sex (Female)	140 (46.5%)	35 (43.8%)	0.660
Self-Reported Race			
White/Caucasian	214 (71.1%)	69 (86.3%)	<0.001
Black/African American	52 (17.3%)	1 (1.3%)	
Other	35 (11.6%)	10 (10.0%)	
Ethnicity (Hispanic/Latino)	45 (15.1%)	10 (12.5%)	0.117
Co-morbidity (Yes)	77 (25.6%)	19 (23.8%)	0.737
Adrenal Insufficiency	7 (2.4%)	3 (3.9%)	0.465
Immunosuppression	33 (11.0%)	9 (11.3%)	0.942
Bone Marrow Transplantation	9 (3.0%)	3 (3.8%)	0.729
PRISM III	11 (6, 16)	11 (6, 17)	0.903
Corticosteroids (Yes)	144 (47.8%)	39 (48.8%)	0.885
Day 1 Hydrocortisone	98 (33.3%)	20 (26.0%)	0.217
Day 1-3 Hydrocortisone	115 (39.1%)	25 (32.5%)	0.284
28-day Mortality	17 (5.7%)	8 (10.1%)	0.158
Complicated Course	69 (23.0%)	18 (22.8%)	0.968

Supplementary Table 3: Multivariable logistic regression testing the association between *PPARγ* SNV rs1801282 and 28-day mortality stratified by corticosteroid exposure.

	Odds Ratio	95% CI	P value
**Corticosteroid Naïve**			
Age (per unit)	0.83	0.58–1.10	0.238
Sex (Female vs Male)	0.16	0.01–1.03	0.105
Race (Black vs White)	0.95	0.04–8.23	0.966
Race (Other vs White)	0.66	0.03–5.90	0.745
Any comorbidity (Yes vs No)	4.32	0.80–23.40	0.080
**rs1801282 mutant (MUT vs WT)**	**1.06**	**0.15–5.53**	**0.952**
**Corticosteroid Treated**			
Age (per unit)	0.91	0.74–1.09	0.330
Sex (Female vs Male)	1.13	0.35–3.61	0.840
Race (Black vs White)	1.38	0.19–6.51	0.706
Race (Other vs White)	1.76	0.24–8.30	0.509
Any comorbidity (Yes vs No)	3.36	1.10–10.77	0.034
**rs1801282 mutant (MUT vs WT)**	**2.04**	**0.53–7.18**	**0.276**

Supplementary Table 4: Cox Proportional Hazards Regression Testing Effect of *PPARγ* **rs1801282** mutant on 28-day survival time stratified by corticosteroid exposure.

	Hazard Ratio	95% CI	P value
**Corticosteroid Naïve**			
Age (per unit)	0.86	0.65–1.14	0.310
Sex (Female vs Male)	0.18	0.02–1.49	0.112
Race (Black vs White)	0.86	0.09–8.47	0.898
Race (Other vs White)	0.80	0.08–7.87	0.851
Any comorbidity (Yes vs No)	2.94	0.63–13.59	0.168
**rs1801282 mutant (MUT vs WT)**	**0.79**	**0.15–4.12**	**0.777**
**Corticosteroid Treated**			
Age (per unit)	0.90	0.75–1.08	0.267
Sex (Female vs Male)	1.39	0.46–4.24	0.563
Race (Black vs White)	1.44	0.30–6.84	0.649
Race (Other vs White)	1.49	0.31–7.18	0.620
Any comorbidity (Yes vs No)	3.37	1.20–9.41	0.021
**rs1801282 mutant (MUT vs WT)**	**2.06**	**0.65–6.51**	**0.217**
